# Implementation of a Synchronized Oscillator Circuit for Fast Sensing and Labeling of Image Objects

**DOI:** 10.3390/s110403401

**Published:** 2011-03-24

**Authors:** Jacek Kowalski, Michal Strzelecki, Hyongsuk Kim

**Affiliations:** 1 Institute of Electronics, Technical University of Lodz, Wolczanska 211/215, 90-924 Lodz, Poland; E-Mail: jackowal@p.lodz.pl; 2 Division of Electronics and Information Engineering, Chonbuk National University, 561-756 Jeonju, Korea; E-Mail: hskim@chonbuk.ac.kr

**Keywords:** synchronized oscillator network, parallel image segmentation, labeling, VLSI CMOS implementation

## Abstract

We present an application-specific integrated circuit (ASIC) CMOS chip that implements a synchronized oscillator cellular neural network with a matrix size of 32 × 32 for object sensing and labeling in binary images. Networks of synchronized oscillators are a recently developed tool for image segmentation and analysis. Its parallel network operation is based on a “temporary correlation” theory that attempts to describe scene recognition as if performed by the human brain. The synchronized oscillations of neuron groups attract a person’s attention if he or she is focused on a coherent stimulus (image object). For more than one perceived stimulus, these synchronized patterns switch in time between different neuron groups, thus forming temporal maps that code several features of the analyzed scene. In this paper, a new oscillator circuit based on a mathematical model is proposed, and the network architecture and chip functional blocks are presented and discussed. The proposed chip is implemented in AMIS 0.35 μm C035M-D 5M/1P technology. An application of the proposed network chip for the segmentation of insulin-producing pancreatic islets in magnetic resonance liver images is presented.

## 1. Introduction

Image object detection is an important stage in image processing and analysis. Usually it is defined in terms of segmentation—a partitioning of the image into separate regions according to rules that somehow describe region homogeneity. Numerous segmentation methods have been developed, but the problem is still unsolved because the enormous variability of image analysis tasks usually requires an individual segmentation approach. A possible solution is to consider a network of synchronized oscillators. This approach is based on the temporal correlation theory, and attempts to describe scene recognition as if performed by a human brain [1]. It has been demonstrated that this network is a reliable tool for the segmentation of textured and biomedical images [2–4]. Other research performed on synchronized oscillatory networks in relation to the human visual system is presented in [5].

Recently, an increasing number of CMOS realizations of image processing devices can be observed [6]. The main objective of designing and manufacturing integrated circuits implementing tools used in image processing is the speed of performed analysis. Even with the fast development of microprocessors and their functionalities, multicore solutions and the increase in computing performance by making use the power of the GPU, the application specific integrated circuits (ASICs) are still outperforming all other available solutions. Thus, the chip that provides very fast image segmentation, practically performed in a real time just after the image acquisition, is a very useful tool with application in many image analysis tasks. One possible field of application is the support of medical diagnosis, e.g., cell counting in large data sets of microscopic histological images [7] or object detection in cross-section images of 3D MRI data [8]. An oscillator network can also be manufactured as a Very Large-Scale Integration (VLSI) chip for very fast parallel image segmentation. We describe a network chip implementing 32 × 32 Synchronized Oscillator Network (SON). A description of an applied oscillator model can be found in [9]. The described network chip allows the analysis of binary images. For example, segmentation and labeling of such images are very important aspects of biomedical image analysis [7,8]. Another realization of an oscillator network chip was presented in [10]. The proposed model is more flexible than the one described in [10] because it provides full dynamic behavior of the oscillator network, as in the original Wang-Terman model [11].

The proposed circuit performs the image segmentation in a real time, resulting in labeled image objects and their number. What is important, and taking into consideration the properties of the oscillator network, it is easy to extend the circuit functionalities by adding more image processing algorithms, e.g., morphological filtering [12] or object boundary detection [3]. These additional functionalities will be considered in the version of the network circuit prototype, after successful testing of the current chip version.

The paper is organized as follows: Section 2 describes a mathematical model of the oscillator and the properties of the network we built using the model. Section 3 presents a CMOS cell structure, an oscillator CMOS circuit, and its Spectre (http://www.cadence.com) simulation results. Also, global inhibitor circuit is described there. Section 4 describes the network chip architecture, and presents our test results for the basic chip building blocks. Also, in this Section, an experimental laboratory setup for image segmentation is shown along with an overview of oscillator tuning. The segmentation results from a sample binary biomedical image using a tuned chip and discussion of our results conclude this Section. Finally, our conclusions are presented in Section 5.

## 2. Mathematical Model of the Oscillator

**Figure 1.**
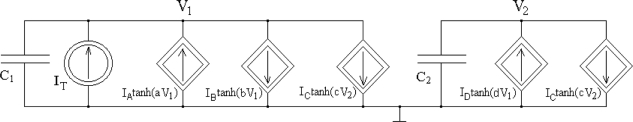
A circuit representation of the mathematical model of an oscillator.

The following mathematical model of an oscillator, which can be physically realized using operational transconductance amplifiers (OTAs), is proposed:

(1) $$\begin{array}{c} C_{1}\frac{{\mathrm{dV}}_{1}}{\mathrm{dt}}=I_{A}\;\text{tanh}\left({\mathrm{aV}}_{1}\right)-I_{B}\;\text{tanh}\left({\mathrm{bV}}_{1}\right)-I_{C}\;\text{tanh}\left({\mathrm{cV}}_{2}\right)+I_{T}\\ C_{2}\frac{{\mathrm{dV}}_{2}}{\mathrm{dt}}=I_{D}\;\text{tanh}\left({\mathrm{dV}}_{1}\right)-I_{C}\;\text{tanh}\left({\mathrm{cV}}_{2}\right) \end{array}$$

where *V_1_* is an excitatory variable and *V_2_* is an inhibitory variable; *I_A_*, *I_B_*, *I_C_*, *I_D_*, *C_1_*, *C_2_* and *a*, *b*, *c*, *d* are constants; and *I_T_* is the total external excitation of a given oscillator. A circuit representation of the oscillator model described by the system of nonlinear differential equations Equation (1) is shown in Figure 1. The presented oscillator model is mathematically known as relaxation oscillator. The oscillation amplitude of the state variable *V_1_* can be expressed as (derivation of this equation is presented in the Appendix):

(2) $$A_{V1}=-\frac{1}{b}\mathrm{arc}\;\text{tanh}\left[\text{tanh}\left(\sqrt{2}\frac{b}{a}\right)-\frac{{2I}_{A}}{I_{B}}\right]$$

Assuming that *A_V1_* = 0.5 V and taking into account the voltage and current limitations of AMIS 0.35 μm technology, the following oscillator parameter values were selected: *I_A_* = 1.2 μA, *I_B_* = 2 μA, *I_C_* = 2 μA, *I_D_* = 2 μA, *a* = 10 V^−1^, *b* = 2.44 V^−1^, *c* = 2.44 V^−1^, *d* = 500 V^−1^, *C_1_* = 50 fF*,* and *C*_2_ = 1.1 pF. Using the analytical short-channel Sakurai-Newton metal-oxide semiconductor field-effect transistor (MOSFET) model [13] with a parameter LAMBDA = 0 and neglecting the body effect, the following expression determines the *I_o_*(*V_r_*) transfer characteristic of the OTA [14]:

(3) $$\begin{array}{c} I_{o}=\begin{array}{c} \{\begin{array}{llll} I_{\mathrm{sat}} & & \mathrm{for} & V_{r}\geq \sigma \\ -I_{\mathrm{sat}} & & \mathrm{for} & V_{r}\leq -\sigma \end{array} \end{array}\\ V_{r}=\sqrt[{n_{r}}]{\frac{\left(I_{\mathrm{sat}}+I_{0}\right)L_{\mathrm{EFFr}}}{2W_{r}B_{r}}}-\sqrt[{n_{r}}]{\frac{\left(I_{\mathrm{sat}}-I_{0}\right)L_{\mathrm{EFFr}}}{2W_{r}B_{r}}}\;\;\;\mathrm{for}-\sigma <V_{r}<\sigma \end{array}$$

where *V_r_* is the differential input voltage of the OTA; *B_r_* is the differential pair MOSFET saturation current factor; *W_r_* and *L_EFFr_* are the width and effective channel length of the differential pair MOSFETs, respectively; *n_r_* is the saturation current coefficient of the differential pair transistors; and *I_sat_* and *σ* are the saturation current and saturation voltage of the OTA, respectively. The transfer characteristic described by Equation (3) can be approximated using the following expression:

(4) $$I_{o}=I_{\mathrm{sat}}\;\text{tanh}\left(n_{r}\cdot 2^{\frac{1-n_{r}}{n_{r}}}\sqrt[{n_{r}}]{\frac{B_{r}W_{r}}{I_{\mathrm{sat}}L_{\mathrm{EFFr}}}}\cdot V_{r}\right)$$

**Figure 2.**
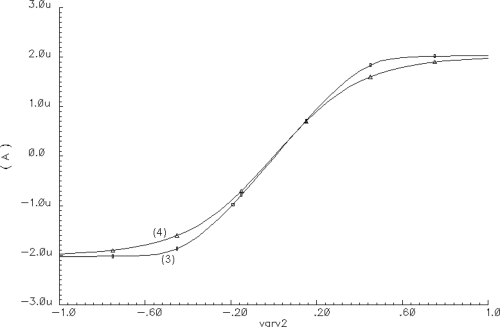
Approximation of Equation (3) by means of Equation (4).

An example of the approximation of function given by Equation (3) by means of Equation (4) is shown in Figure 2. For a SON, the total external current excitation *I_T_* of each oscillator is defined as

(5) $$I_{T}=I_{\mathrm{out}}+I_{F}\overset{4}{\underset{j=1}{\cup }}\mathrm{Hev}(V_{1}{}^{j})-I_{\mathrm{GI}}\mathrm{Hev}(\mathrm{GI})-I_{E}$$

where *I_out_* is an output current from the input circuit. In addition, *I_out_ =* 1 μA for image objects, *I_out_ =*−1 μA for image background, *I_F_* = 0.8 μA is the current of weights polarization, *I_GI_ =* 0.22 μA is the current of the global inhibitor polarization, *I_E_* = 2.5 μA is the constant polarization current required for proper operation of the oscillator circuit, *V*_1_*^j^* is the output V1 voltage of the j-th neighbor cell, GI is the output of the so-called global inhibitor circuit (see description GI in Section 4), which receives information from oscillators and in turn eventually can inhibit the whole network, *Hev* is the Heaviside step function and ∪ represents a logical disjunction. The global inhibitor ensures that only one oscillator group (representing a given image object) is activated at a time. A proper selection of the preceding parameter values maintains control of the network oscillators and provides appropriate synchronization and desynchronization.

## 3. CMOS Cell with Weights Circuit

**Figure 3.**
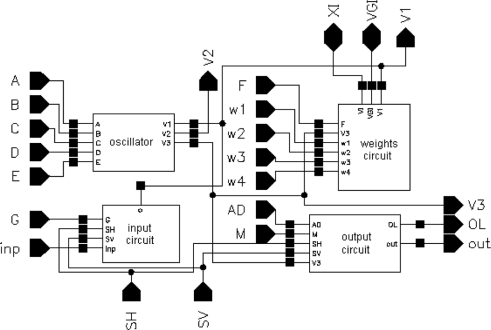
Block diagram of the SON cell.

A block diagram of a cell of the SON is presented in Figure 3. A cell of the oscillatory neural network is composed of the following circuits: input and output, CMOS oscillator, and excitatory synapse (network weights).

**Figure 4.**
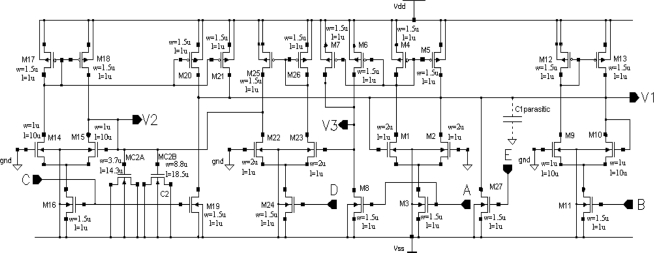
Schema of the oscillator circuit.

Using a circuit representation of the oscillator mathematical model shown in Figure 1, we designed a CMOS circuit OTA-based structure. The schema of the oscillator CMOS circuit is presented in Figure 4. Transistors M1 to M5 realize the function *I_A_* tanh(*aV*_1_), transistors M9 to M13 realize the function *I_B_* tanh(*bV*_1_), transistors M14 to M21 realize two functions *I_C_* tanh(*cV*_2_), transistors M6 to M8 and M22 to M26 realize the function *I_D_* tanh(*dV*_1_), and transistor M27 is the current source *I_E_*. The dimensions of the transistors have been designed to ensure that all MOS transistors always work in the saturation region for the assumed oscillation amplitudes. To save silicon area and to use one poly technology, a capacitor C_2_ was implemented using the gate capacitances of transistors MC2A and MC2B. Because the channels of these transistors work continuously in strong inversion, the equivalent capacitance of this structure is linear for the assumed oscillation amplitudes of state variables V_1_ and V_2_. A capacitor C_1_ was implemented by a sum of parasitic layout capacitances, C1_parasitic_ (Figure 4). The oscillator layout occupied a 51 μm × 32.5 μm (1,657 μm^2^) silicon area. With a typical supply voltage of 3.3 V, the supply current is about 12 μA, so power consumption by the oscillator is about 40 μW.

**Figure 5.**
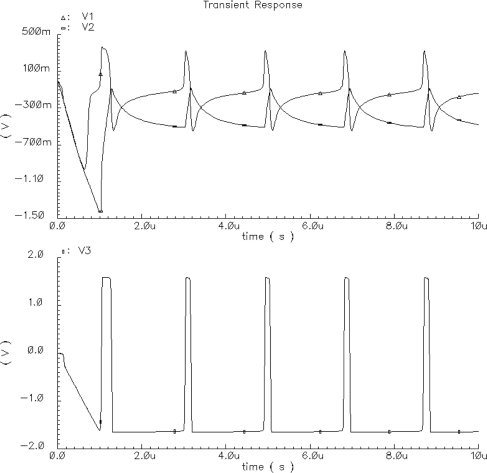
Simulated waveforms of oscillator voltages V_1_(t), V_2_(t), V_3_(t) for I_T_ = −I_E_ = −1.5 μA.

The Spectre software transient simulation results of the oscillator voltage waveforms V_1_, V_2_, and V_3_ are shown in Figure 5. The voltage waveform V_3_ is a binarized voltage V1 with a threshold equal to zero. The simulation was performed using the BSIM3v3.2 Level 53 MOS transistor model, taking all layout parasitic capacitances into consideration.

**Figure 6.**
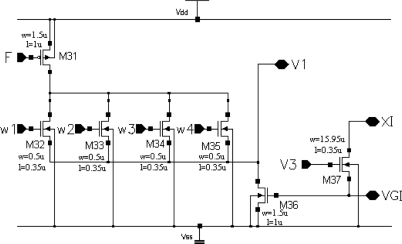
A schema of the excitatory synapse circuit.

A schema of the excitatory synapse circuit is shown in Figure 6. Transistor M31 works as a current source with typical value I_F_ = 0.8 μA, the current of which is switched by transistors M32–M35 to node V1 of the given oscillator. Gates of transistors M32–M35 are connected to V3 nodes of the four neighbour cells. M36 transistor plays a role of a current mirror, which reflects a state of a global inhibitor (node VGI in Figure 6). M37 transistor activates the state of global inhibitor VGI during a cycle of oscillations of any cell in the SON.

## 4. A SON Chip Architecture

**Figure 7.**
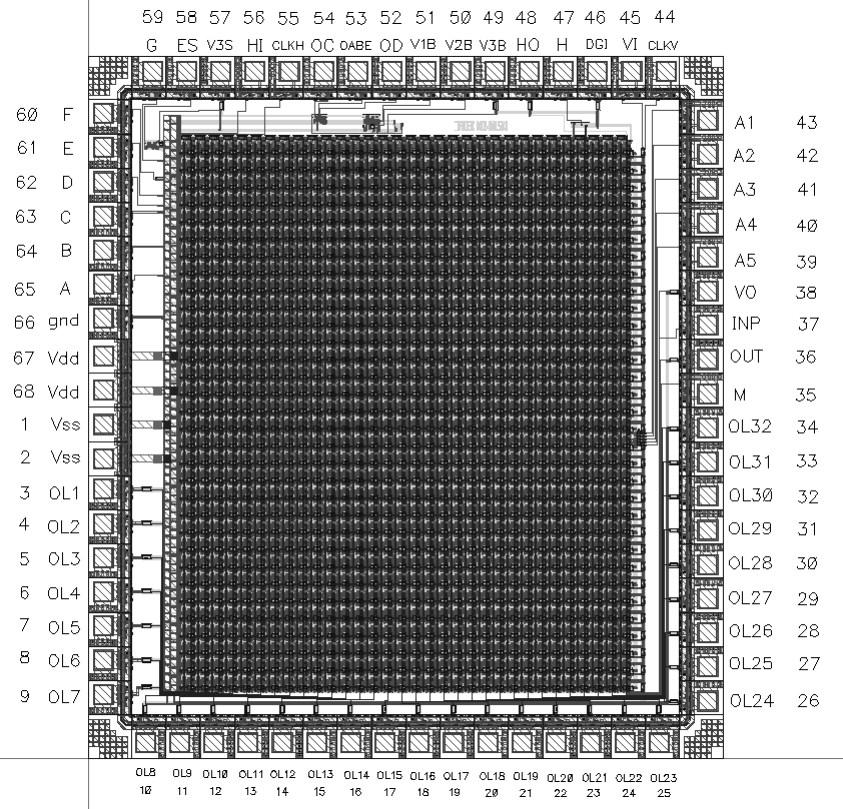
A layout of SON chip.

**Figure 8.**
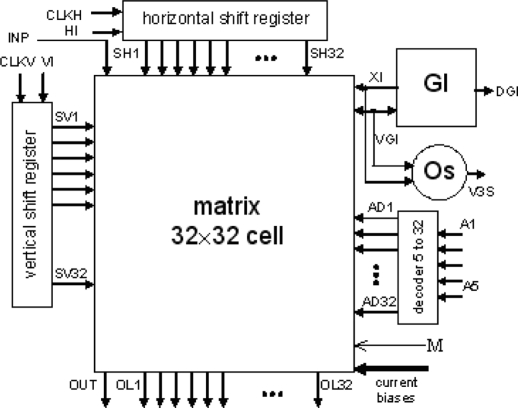
A block diagram of the SON processor.

The layout of a SON chip is shown in Figure 7. It was designed using Cadence software. The chip consists of 90952 MOS transistors and occupies 7.9 mm^2^ of silicon area (2.670 mm × 2.958 mm). Its core without pads has dimensions of 2.3 mm × 2.6 mm. It was encapsulated in a JLCC68 package. The supply current, as measured during image processing, is about 13 mA. The supply voltage is equal to 3 V; thus, the power consumption is about 39 mW. The chip works properly with supply voltages from 2.4 V to 3.6 V. A block diagram of the SON processor is shown in Figure 8.

**Figure 9.**
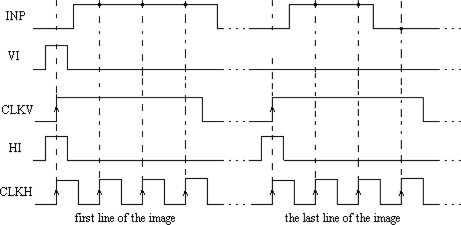
Control signals required for input image loading into a chip.

The main element of the chip is a matrix of 32 by 32 cells that process the image pixels. Each cell consists of an oscillator CMOS circuit, an excitatory synapse (network weights), and input and output circuits. An image is fed into the network chip by serial input INP, pixel-by-pixel and line-by-line. Cells are addressed by horizontal and vertical shift registers. The shift registers are controlled by two clocks: CLKH and CLKV. Signals HI and VI are used to synchronize image loading. Control signals are required to write the input image pixels into a chip, as shown in Figure 9.

A global inhibitor (GI) circuit is connected to all oscillators in the network. This circuit uses two signals: VGI and XI. Node VGI is activated when at least one oscillator in the network is active. The DGI output signal is a binarized version of VGI and is used for observation of the global inhibitor circuit state. Line XI is used to inhibit all oscillators when GI is active. An additional oscillator, connected to the whole network by weights, is connected to GI only. The oscillator Os was implemented to synchronize the operation of the oscillator network and allows counting the number of recognized image objects. V3S is an output signal of this oscillator. The segmented image objects can be output by a serial digital signal, OUT. This output is controlled in the same way as the INP input by horizontal and vertical registers.

It is also possible to observe an activity of a selected oscillator’s row of the network. The chosen row is addressed by a 5 to 32 decoder. Then, the oscillator’s states are available in digital OL1-OL32 outputs. During our network chip testing, a latter technique (selection of oscillators’ rows) was used for observation and analysis of oscillator output. Thus, the network chip is a mixed signal analog-digital circuit. The global inhibitor and additional oscillator circuits are fully analog. Matrix of 32 by 32 cells containing oscillators and its additional circuits are mainly analog, and the shift registers and line decoder are mainly digital circuits.

### 4.1. Tests of Basic SON Chip Building Blocks

**Figure 10.**
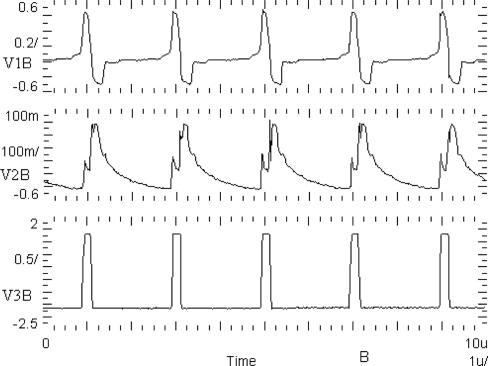
Oscillograms of V1B, V2B, V3B waveforms of CMOS oscillator structure.

A basic building block of the chip is the CMOS oscillator circuit. A schema of the oscillator circuit is shown in Figure 4. The oscillator circuit has been implemented into the chip in the form of a separate test structure. Oscillograms of CMOS oscillator structure waveforms are shown in Figure 10. V1B is an excitatory variable of the oscillator, V2B is an inhibitory state variable, and V3B is a binarized V1B voltage with a threshold equal to zero. These waveforms are correct and agree with computer simulations (Figure 5) performed using Spectre software during the chip design.

### 4.2. Laboratory Setup for Image Segmentation

To perform image segmentation, an experimental laboratory setup was constructed based on the work in [15]. Our setup consisted of the following elements:

- A personal computer (PC);
- a universal I/O PCI card (National Instruments NI PCI 7831R), and
- a special module containing an integrated circuit of the oscillator network.

A PC computer working under Microsoft Windows XP and LabView software (ver. 7.1) was used to program the NI PCI 7831R card. The I/O card has 96 digital reconfigurable inputs/outputs with an operating frequency of 40 MHz. This card also contains an internal FPGA structure. Ten card connectors were configured as outputs and were used to input a binary image to the network chip. The I/O card was connected to a special external test module that contained a chip with an oscillator network. This module also has I/O buffers for input and output data, and row addresses, and circuits designed to control the chip. Polarization currents were used to set the oscillator network weights and the weight of the GI circuit, and to control other oscillator parameters such as a filling ratio of the waveforms representing an excitatory and an inhibitory state variable. It was also possible to switch additional oscillator (Os) circuits and global inhibitor (GI) circuits on and off.

The LabView code was used to edit, store, and read a sample binary image from a hard disk. Next, the image was uploaded to the network chip. Image pixels were serially transferred to the chip using synchronization signals HI and VI (Figure 9). Due to the charge leakage in CMOS structures, the image needed to be loaded repeatedly. The refreshing time was controlled by the LabView software. This software was stored inside the FPGA structure and allowed the setting of the refreshing period starting from 1 ms in steps of 1 ms. Measurements of chip parameters showed that the refreshing period should be shorter than 4–5 s. However, this exceeds the image analysis time (see Section 4.3), thus there is no need to refresh the loaded image to obtain a correct segmentation. After an image was loaded into the network, the oscillators began to oscillate. The oscillator output was obtained for each row, as addressed by the software. Another row could be observed only after a change of the address performed by the I/O card. An appropriate setting of polarization currents that controlled network weights and the GI weight allowed us to find a stable network state where oscillators connected to a given image object were in synchrony, and where oscillator groups representing different objects were desynchronized.

### 4.3. Oscillator Tuning

During our first functional chip tests, some problems with long chain object oscillator synchronization occurred. These problems were caused by a mismatch in oscillation periods [10,16] in the network. Since the oscillator circuits are analog, a mismatch of MOS transistors caused a mismatch in oscillation frequencies. Some synchronization improvement can be achieved by increasing the power of excitation synapse weights, which can be realized by increasing current I_F_ in the chip. Nevertheless, this method is sometimes ineffective because it causes problems with the desynchronization of oscillator groups connected to different objects. It appears that the best solution is tuning all oscillators to one frequency. To adjust the free frequencies of oscillators in the network, it should be possible to tune each oscillator separately (tuning would affect the oscillator parameters that influence its frequency).

Since there are many oscillators in the network, the implementation of separate tuning circuits and tuning mechanisms can consume silicon area and add cost. Therefore, combining image inputting into the network with oscillator tuning seems to be the best solution. The tuning procedure can be realized using analog properties of an input circuit in the oscillator cell. When an image is loaded without tuned oscillators, the current I_out_ =I_G_ = 1 μA for image objects, and I_out_ = −I_G_ = −1 μA for the image background. A transitional part of the static transfer characteristic of the input circuit has not been used thus far, but it can be used for tuning oscillator frequencies. When an image is loaded with tuned oscillators, the transitional part of the static transfer characteristic of the input circuit is used. Thus, *I_out_* = *I_G_* tanh {*g*(*V_in_* – 1.5 V)} for image objects, and *I_out_ =* −*I_G_* = −1 μA for the image background. Consequently, the image pixel voltage value *V_in_* for the image object can be tuned to obtain equal free frequencies in all oscillators. The maximum current *I_out_* tuning range is from 1 μA to −1 μA, which corresponds to the effective input voltage *V_in_* from 2 V to 1 V. For an image background, *V_in_* = 0 V. We describe tuning matrix K as follows:

(6) $$K=\left[\begin{array}{cccc} k_{11} & k_{12} & ... & k_{1\;32}\\ k_{21} & k_{22} & ... & k_{2\;32}\\ ... & ... & ... & ...\\ k_{32\;1} & k_{32\;2} & ... & k_{32\;32} \end{array}\right]$$

Then, assuming that the maximum voltage *V_in_* = 3 V, we have the following relation for image objects:

(7) $$I_{\mathrm{out i,j}}=I_{G}\;\text{tanh}\left\{g\left(3V\cdot k_{\mathrm{ij}}-1.5V\right)\right\}\;\;\;\;\;\;\;i,j=1...32$$

The oscillator tuning procedure is described as follows:

Set oscillator currents *I_F_ = 0* and *I_H_ = 0* to obtain oscillation without excitatory synaptic connections and without global inhibitors (free oscillations). All oscillators in the network should oscillate. If this is not the case, slightly decrease current *I_E_*.

Assuming *k_ij_ =* 1 for *i,j* = 1 ,..., 32, find the oscillator with the smallest frequency *f_s_* of free oscillations.

Step-by-step, tune the *k_ij_* coefficient for each oscillator in the network to have all oscillator frequencies as close to frequency *f_s_* as possible.

**Figure 11.**
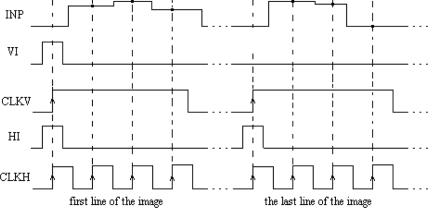
Control signals required for input image loading into a chip with oscillators frequency tuning.

The precision of image segmentation by SON depends on the precision of the aforementioned tuning procedure performance. The control signals required for writing the input image pixels into a chip with oscillator frequency tuning are shown in Figure 11.

### 4.4. Analysis of a Sample Biomedical Image Using the Tuned SON Chip

**Figure 12.**
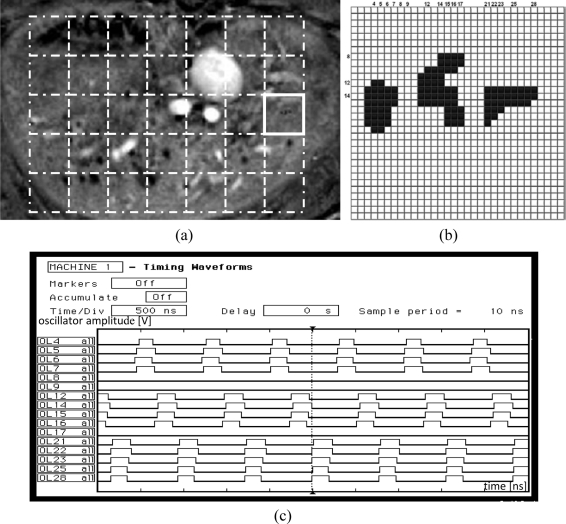
Sample MR image of rat liver with pancreatic islets **(a)**, dashed squares mark regions for network chip labeling. Sample region loaded into a chip **(b)**, marked by solid line in (a). Selected oscillators output waveforms (defined by column numbers in b) for line 14 **(c)**.

In this section, the results of a sample binary biomedical image segmentation using the tuned network chip are presented. An automatically tuned chip was used for image segmentation. After successful automatic tuning using the LabView procedure, the chip was tested on the labeling of insulin-producing pancreatic islets in magnetic resonance (MR) images. Such islets were transplanted into a rat liver. This technique is used to cure Type 1 diabetes [8]. To evaluate the success of transplantation, the number of active islets should be counted. A sample MR image of the rat liver is shown in Figure 12(a). Active pancreatic islets are visible as dark spots.

To perform segmentation and islet labeling, the liver region was divided into non-overlapping windows of size 32 × 32 which correspond to the network matrix, as shown in Figure 12(a) (dashed line squares). Next, after local thresholding, each region was analyzed separately by the oscillator network. Each detected object in any image was labeled using a unique value. Finally, the borders of each region were investigated to determine whether some of the detected islets split into different regions. In this case, the labels that described different fragments of the same islet were converted into a single label.

The latter operation was performed off-chip. The network operation is illustrated on a sample region, marked by a bold square in Figure 12(a). An image corresponding to this region, loaded into the network chip, is shown in Figure 12(b). After loading the image, the segmentation was performed by obtaining oscillator synchronization within any object, and oscillator group desynchronization between oscillators belonging to different object groups. For example, sixteen selected oscillator waveforms for line 14 are presented in Figure 12(c).

The analyzed image fragment contains three objects thus oscillators 3–7, 11–17, and 21–28 which represent these objects are in synchrony. Due to the limitations of a logic analyzer usind for chip analysis, in the Figure 12(c) only selected of corresponding oscillator waveforms are shown, e.g., for oscillators 4, 5, 6, 7 (object #1); 12, 14, 15, 16 (object #2), and 21, 22, 23, 25, 28 (object #3) respectively. In fact, these three oscillator groups oscillate with a phase shift (as they represent different objects). As a consequence, waveform analysis of these oscillators, and also the waveforms of oscillators from all other lines (not shown in Figure 12(c)), allows the detection and labeling of each object (image object is represented by all oscillators activated in a given time). Oscillators that belong to the image background do not oscillate (e.g., oscillators 8, 9, 17, Figure 12(c)). The complete image segmentation occurs when all oscillator groups representing different objects were activated and oscillated. This process takes place in one period of an active oscillator. Thus the segmentation time corresponds to this period length and it is about 1 μs in the case of the example presented in Figure 12(b).

After analysis of each region, the image data from the next fragment is loaded into the network chip and entire process is repeated. The labeling time of the image fragment shown in Figure 12(a) (224 × 160 pixels) depends of the number of regions, and in this case is equal to 35 × (1 μs + 8 ms) = 280 ms (the image load for each region takes 8 ms and depends on a universal National Instruments I/O PCI card). Thus, the image loading procedure is the most time consuming procedure, and dominates the time period required for segmentation. Also, the proposed SON architecture leads to a much faster segmentation than that obtained by the chip described in [10], where the analysis of a 16 × 16 region took from 32 to 142 μs.

### 4.5. Discussion

The segmentation time of analyzed sample images is about 1 μs, which does not depend on image size, but only on the number of image objects. The maximum number of objects, which can be recognized (segmented) by the circuit, depends on the ratio between the whole period length and the time of the active phase. These two parameters depend on circuit polarization currents. The applied CMOS circuit manufacturing technology defines currents range and their maximum value. When the number of objects is increasing, the ratio of the oscillator silent phase and the active phase must also be increased. The duration of the silent phase must be equal to the time of the active phase multiplied by the number of image objects [17]. Thus, the larger object number leads to a longer oscillation period and to an increasing segmentation time. Nevertheless, the time is much shorter if compared to computer simulations, as shown in Table 1. It presents analysis times for the image from Figure 12(a) when network chip and PC computer (P4, 3 GHz) were applied, respectively.

In the computer simulations, segmentation of the single segment requires 0.24 s, and 8.57 s is required for the whole image. The performance comparison is performed for a single image segment and the entire image. Also, the segmentation time of a 16 × 16 image for a network chip described in [10] is shown. For larger images, the analysis time increased due to the necessity of loading new segments into the chip. This load time is relatively long and depends on the I/O interface between the SON chip and the PC. Optimizing the FPGA architecture of the I/O card could reduce the time by providing a much faster segmentation of the whole image. It also can be seen from Table 1 that the proposed SON architecture leads to much faster segmentation than that obtained by the chip described in [10], even considering that the latter represents a matrix with only 16 × 16 nodes. Main advantages of proposed circuit to the digital approaches are high speed, low power consumption and relatively small chip area. There are no many analog processors for image segmentation reported in the literature. One of these works is presented in [18], where the circuit performs image analysis by detection of object contour. However major drawbacks of this design are that it can only recognize centered figures and it cannot detect figures with large gaps in their contour. The circuit presented in [10] also implements the oscillator network, however test results shows that our chip is faster, it has larger matrix size (32 × 32 compared to 16 × 16) and expresses lower power consumption (38 μW compared to 76 μW). The most time consuming step is the oscillator tuning procedure. However, this procedure occurs only once for a given chip during its initial tests. Thus, it does not influence the image segmentation time.

## 5. Conclusions

A new CMOS chip implementing a SON with a matrix size of 32 × 32 was presented. The chip was implemented in AMIS 0.35 μm C035M-D 5M/1P technology and is able to perform fast segmentation of binary images. The proposed chip architecture enables serial loading of the input image and reading of the output segmented images. In addition, it is also possible to observe the activity of the addressed oscillator line. It was demonstrated that the VLSI CMOS SON chip provided appropriate operation (oscillator synchronization within an object, and oscillator group desynchronization between different objects).

To speed up the network operation, oscillators connected to the image background should not oscillate. When compared to the solution presented in [10], the SON does not assume the image background to be an object. Furthermore, the approach of implementing an additional oscillator connected only to the global inhibitor was successful. The analysis of output waveforms of this oscillator allowed us to find the start and stop time instants of the image segmentation process (segmentation of the whole image occurred between the neighbor active states of the oscillator).

The period variability of the oscillator outputs caused by a technological mismatch of MOS transistor parameters is a main disadvantage of the proposed network chip. The variability caused differences in the filling ratios of oscillator outputs, resulting in problems with their synchronizations and subsequent difficulties in the detection of a larger number and length of objects. This highly undesirable phenomenon has been effectively compensated by the use of the proposed automatic oscillator tuning procedure. It results in an increment of a number of the image objects that can be segmented by the network chip. The network can simultaneously analyze four objects. Considering a small matrix size (32 × 32), this might be sufficient for segmentation of some real-life image fragments. It is possible to increase the matrix size (e.g., to 256 × 256) by the manufacturing of the presented network chip using modern analog technology, like UMC 90 nm and 65 nm CMOS mixed/RF process. The proposed oscillator network chip was tested on sample biomedical images (like rat liver MR images discussed in Section 4.4). The test results are promising; in the case of the analysis performed all image objects were correctly detected. However, a number of further tests are needed to prove the full chip functionality, which allow its implementation to support pathological or medical diagnosis.

## Figures and Tables

**Figure 13.**
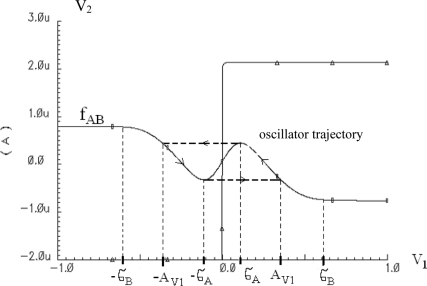
Nullclines and trajectories (dashed line) for single relaxation oscillator.

**Table 1.** Analysis times of image from Figure 12(a) using different approaches.

	
	**32 × 32 segment**	**Whole image (224 × 160)**
**Computer simulation**	0.24 s	8.57 s
**SON ASIC**	1 μs	280 ms
**16 × 16 chip presented in [10]**	142–38 μs	-

## Appendix

Expression for the oscillation amplitude A_V1_ of the state variable *V_1_* is required for CMOS oscillator circuit design. It allows the calculation of all oscillator parameters for assumed oscillation amplitude *A*_*V*1_. From Equation (1) and Figure 13:

$$f_{\mathrm{AB}}=I_{A}\;\text{tanh}\left({\mathrm{aV}}_{1}\right)-I_{B}\;\text{tanh}\left({\mathrm{bV}}_{1}\right)+I_{T}$$

When we consider nullclines and trajectories for single relaxation oscillator as shown in Figure 13, then:

$$\begin{array}{c} f_{\mathrm{AB}}\left(\sigma_{A}\right)=f_{\mathrm{AB}}\left(-A_{V1}\right)\\ I_{A}\;\text{tanh}\left({a\sigma }_{A}\right)-I_{B}\;\text{tanh}\left({b\sigma }_{A}\right)=I_{A}\;\text{tanh}\left(-{\mathrm{aA}}_{V1}\right)-I_{B}\;\text{tanh}\left(-{\mathrm{bA}}_{V1}\right)\\ I_{A}\;\text{tanh}\left({a\sigma }_{A}\right)≅I_{A}\\ I_{A}\;\text{tanh}\left(-{\mathrm{aA}}_{V1}\right)≅-I_{A}\\ I_{A}-I_{B}\;\text{tanh}\left({b\sigma }_{A}\right)=-I_{A}-I_{B}\;\text{tanh}\left(-{\mathrm{bA}}_{V1}\right)\\ I_{B}\;\text{tanh}\left(-{\mathrm{bA}}_{V1}\right)=I_{B}\;\text{tanh}\left({b\sigma }_{A}\right)-{2I}_{A}\\ A_{V1}=-\frac{1}{b}\mathrm{arc}\;\text{tanh}\left[\;\text{tanh}\left({b\sigma }_{A}\right)-\frac{{2I}_{A}}{I_{B}}\right] \end{array}$$

because:

$$\sigma_{A}=\frac{\sqrt{2}}{a}$$

then finally:

$$A_{V1}=-\frac{1}{b}arc\;\;\text{tanh}\left[\text{tanh}\left(\sqrt{2}\frac{b}{a}\right)-\frac{{2I}_{A}}{I_{B}}\right]$$
